# High Sensitivity Online Sensor for BTEX in Ambient Air Based on Multiphoton Electron Extraction Spectroscopy

**DOI:** 10.3390/s25144268

**Published:** 2025-07-09

**Authors:** Uriah H. Sharon, Lea Birkan, Valery Bulatov, Roman Schuetz, Tikhon Filippov, Israel Schechter

**Affiliations:** Schulich Faculty of Chemistry, Technion-Israel Institute of Technology, Haifa 32000, Israel; uriahsharon@campus.technion.ac.il (U.H.S.); slbirkan@campus.technion.ac.il (L.B.);

**Keywords:** gas-phase MEES, multiphoton electron extraction spectroscopy, trace gas analysis, electronic nose (e-nose), gas trace detection, environmental monitoring, VOC, BTEX

## Abstract

Benzene, toluene, ethylbenzene, and xylene (BTEX) are widespread volatile organic compounds commonly present in fuels and various industrial materials. Their release into the atmosphere significantly contributes to air pollution, prompting strict regulatory concentration limits in ambient air. In this work, we introduce Multiphoton Electron Extraction Spectroscopy (MEES) as an innovative technique for the sensitive, selective, and online detection and quantitation of BTEX compounds under ambient conditions. MEES employs tunable UV laser pulses to induce the resonant ionization of target molecules under a high electrical field, with subsequent measurement of the generated photocurrent. We now demonstrate the method’s ability to detect BTEX in ambient air, at part-per-trillion (ppt) concentration range, providing distinct spectral signatures for each compound, including individual xylene isomers. The technique represents a significant advancement in BTEX monitoring, with potential applications in environmental sensing and industrial air quality control.

## 1. Introduction

Sensitive detection and quantification of volatile organic compounds (VOCs) is a necessity in a variety of disciplines. VOCs were found to have a wide range of negative effects on both the environment and human health. Environmentally, VOCs contribute to material degradation and oxidative processes, as well as exacerbate global warming, primarily by altering ozone concentrations. In terms of health, the liver, heart, kidneys, skin, and lungs are some examples of the organs that may be negatively affected by exposure to VOCs. Furthermore, VOC exposure has been linked to increased risks of diseases including diabetes, cancer, asthma, and allergies [1].

Among VOCs, the group comprising benzene, toluene, ethylbenzene, and xylene—collectively referred to as BTEX—has garnered significant attention in environmental research due to its high prevalence and toxicity. All four compounds are listed as Hazardous Air Pollutants (HAPs) [2,3,4,5]. BTEX research is world-encompassing and ongoing [3,4,5,6,7]. High concentrations of BTEX in air are a product of the myriad industrial and domestic processes. Ambient BTEX levels are primarily driven by fuel evaporation and combustion [3,4,5,8], with additional industrial contributions stemming from oil transportation and solvent use [4,5,8]. Other sources of BTEX pollution include biological degradation and accidental industrial releases [5,9]. Atmospheric pollutants are then distributed around the world by meteorological conditions [5]. Indoors, BTEX pollution is sometimes even higher than in ambient air, and can be created by seemingly harmless day-to-day activities, like home warming [4], stove-top cooking [6], writing using whiteboard markers [10], or using cleaning products [8].

The health risks associated with BTEX exposure are substantial and numerous. BTEX compounds can be absorbed into the human body via inhalation, ingestion, and dermal penetration [5]. Benzene is a well-established carcinogen, while ethylbenzene is classified as a possible carcinogen [3,5,10]. BTEX exposure can also negatively influence the hematological, lymphatic, immune, reproductive, respiratory, and nervous systems. More localized effects of BTEX include kidney and liver damage as well as hair loss [3,5]. As VOCs, BTEX also contributes to global warming [3]. 

Given these risks, the necessity of BTEX concentration monitoring is undeniable. Various governmental and international organizations have established recommended exposure limits. The World Health Organization (WHO) has set the reference value to 0.05 ppb (0.17 μg$/m^{3}$) for benzene, 5 ppm (22 $mg/m^{3}$) for ethylbenzene, 200 ppb (870 μg$/m^{3}$) for total xylene isomers, and no specified value for toluene concentration was set [11]. Some of the strictest reference values include 0.04 ppb (0.13 μg$/m^{3}$) for benzene in Massachusetts, 55 ppb (240 μg$/m^{3}$) for ethylbenzene in the Netherlands, 17.6 ppb (80 μg$/m^{3}$) for toluene in Massachusetts, which alsch also specifies an annual limit of 2.7 ppb (11.8 μg/m^3^) for total xylene isomers [12]. It was recently shown that long-term exposure to ambient BTEX, even at levels below current regulatory limits, is associated with increased overall and site-specific cancer risks, highlighting the urgent need for improved methods for detection and monitoring of BTEX compounds in air [13].

A wide array of analytical techniques has been developed for BTEX monitoring in ambient and indoor air across rural, industrial, and urban settings. For broad applicability, analytical methods must support a wide concentration range [4]. For accurate results, many methods require specific sampling and preparation methods as a preliminary step, to remove interferences, and adapt the sample to the detection method [4]. 

The most common analytical method used today for BTEX analysis is gas chromatography (GC), coupled to a variety of detectors [4,11,14,15,16]. GC is favored for its high accuracy and the fact that no reagents are needed for its usage; however, GC results are often less than satisfactory in the presence of complex matrices, and the separation and detection method is time-consuming. 

The flame ionization detector (FID) is often used in gas chromatography of BTEX. It provides universal, rapid, and stable organic compound detection but lacks selectivity and requires three gas sources and precise airflow control. Photoionization detectors are also used. They offer fast detection down to 1 ppb with broad linear range and compact instrumentation; however, their performance can degrade in high-humidity conditions [4]. Mass spectroscopy (MS) is often used for the detection of BTEX, with LODs in the ppt range, good selectivity, precision, and a large dynamic range. Still, GC-MS has high management costs, is challenging to operate in real-time, and may perform poorly at high analyte concentrations [4,16]. 

Other BTEX analysis approaches include sensor-based platforms [17], additional MS techniques, and various spectroscopies. Sensors can detect BTEX at ppm to ppb levels and offer portability and simplicity, though they generally lack the sensitivity and specificity of full-scale analytical instruments. They often require precise environmental conditions and are not very reliable [4,16,18]. Ion mobility spectrometry (IMS) is an additional approach that achieves LODs as low as 0.7 ppb in air. It has good linearity and can be portable. However, most IMS setups suffer from low sensitivity and limited molecular specificity [7]. Atmospheric pressure chemical ionization mass spectrometry (APCI-MS) is simple, quick, and provides real-time BTEX monitoring without sample pretreatment, with LODs in the 0.3 ppb (1–2 μg/m^3^) range. Despite its advantages, APCI-MS exhibits limited reproducibility and quantitative reliability [4]. 

Optical spectroscopies have also been applied for BTEX detection [19,20]. Fluorescence spectroscopy can be used for isomer detection; however, it is susceptible to interference by other ions, with poor linearity [16]. UV spectroscopy is relatively rapid, portable, and affordable, though to reach sensitivity as low as 2.5 ppb requires a long sampling path. It also requires an inert gas supply, which impairs the portability of the device, and the determination of individual BTEX molecules is not possible [18].

Multiphoton ionization (MPI) is a nonlinear optical process in which multiple photons from a short laser pulse are simultaneously absorbed by a molecule, resulting in its ionization. This ionization can proceed via resonant or non-resonant pathways. In resonant ionization, photon energies align with electronic or vibronic transitions in the target molecule, enhancing ionization probability. In contrast, non-resonant ionization lacking such alignment has significantly less probability to take place. Consequently, MPI predominantly takes place using resonant wavelengths, so that measuring ionization in different wavelengths will generate ionization spectra that are characteristic of individual molecules [21,22,23].

Traditionally, MPI has been used in analytical chemistry in several ways, most commonly in conjunction with MS, enabling detailed molecular and fragmentation analyses. Most MPI-MS systems utilize single-wavelength lasers [21,24], though variants such as resonance-enhanced MPI and dual-laser excitation schemes have also been documented. These configurations restrict ionization to a confined focal region, inherently limiting the number of analyte molecules that can be effectively ionized. Moreover, MPI-MS typically requires high vacuum levels to ensure sufficient ion-free paths [23,25]. A partial mitigation strategy has been introduced in previous works, involving the use of an unfocused laser beam combined with time-resolved detection of mirror charges. In this setup, ions generated along the laser path migrate toward a nearby negatively charged electrode. The short ion drift distance (approximately 1 cm) reduces the vacuum requirement to 5 mTorr, which is sufficient to prevent collisional interference. Due to the brief ion transit time, conventional time-of-flight analysis is impractical. Instead, ion detection is achieved by monitoring the transient mirror charges induced in the electrodes. By collecting multiple data points throughout the ion trajectory, this method partially compensates for the reduced resolution inherent in the shorter flight path. While this approach enables the ionization of a significantly larger number of molecules, it compromises molecular specificity, rendering it suitable only for the analysis of relatively simple mixtures [21,23].

Efforts have also been made to apply MPI under ambient atmospheric conditions for analysis of solids and liquids using single-wavelength laser excitation. While this approach demonstrated good sensitivity for a range of molecules, it lacked spectral resolution and thus could not support compound identification [25,26,27].

To address these limitations, we have previously proposed a new analytical platform: Multiphoton Electron Extraction Spectroscopy (MEES). MEES represents a paradigm shift in MPI-based analysis, enabling the direct analysis of solid and liquid samples under ambient conditions. This technique utilizes a pulsed ultraviolet (UV) laser to excite the analyte via a multiphoton mechanism, followed by photoelectron extraction and detection. Compared to ion-based detection in MS, electron detection in MEES is simpler, more accurate, and does not require vacuum systems. The generated photoelectrons are accelerated using a high electric field towards an electrode, where the resulting time-resolved current is integrated to determine the total emitted charge [28,29,30]. The measured photo charge, plotted as a function of laser wavelength, yields a rich, high-resolution spectrum. MEES spectra consist of numerous sharp peaks corresponding to resonant electronic and vibronic energy transitions of the target molecule, enabling molecular fingerprinting. Consequently, MEES yields a far greater number of spectral features than traditional absorption or fluorescence spectroscopy, including transitions to higher excited states that are often inaccessible to single-photon techniques. The resulting high density of spectral peaks substantially enhances the analytical information content, improving molecular specificity and enabling the deconvolution of complex mixtures [21,31].

The exceptional sensitivity of MEES arises from four primary advantages: (1) efficient resonant ionization, (2) reduced recombination probability in high electrical fields, (3) near-complete charge collection enabled by high electrical fields, and (4) low-noise signal acquisition based on electrical current measurement rather than light intensity detection. Signal amplification is achievable with minimal background noise, and the detection limit is governed primarily by the quality of the electronic instrumentation employed.

MEES have been extensively validated for applications in the surface analysis of liquids and solids. Under ambient conditions, detection limits in the fmol range have been achieved, underscoring the technique’s extraordinary sensitivity. In addition to sensitivity, surface-based MEES is distinguished by its high spectral resolution and density of sharp features, enabling effective compound fingerprinting and molecular identification. Comparative analyses have demonstrated that MEES surpasses conventional optical spectroscopies in both sensitivity and information richness [21,28,29,30,31].

In our previous work, we introduced gas-phase MEES, the first implementation of this technique for volatile and semi-volatile compound analysis. Our results revealed the technique’s unique capabilities in the real-time, high-resolution detection of trace-level organics, applicable to fields such as environmental monitoring, homeland security, and medical diagnostics [23]. Continuous gas flow eliminates the need for sample refreshing. The technique demonstrated linear responses over wide concentration ranges and sub-ppb LODs. We have also presented a full spectrum of Benzene, with a LOD of 20 ppt.

In this work, we demonstrate the full scope of BTEX detection available when using the gas-MEES system. Utilizing the gas-MEES for BTEX detection presents us with a device capable of real-time, online monitoring of all BTEX compounds. Each BTEX molecule exhibits a distinct spectral signature, enabling individual identification. The MEES system sensitivity also enables the quantification of concentrations at sub-ppb levels for all BTEX compounds. Furthermore, BTEX detection and quantification are feasible even when mixed. MEES spectra are additive, and an analysis of a mixture is possible without employing separation techniques. The results demonstrate the potential of the gas-phase MEES for analytical applications, particularly in environmental monitoring.

## 2. Materials and Methods

### 2.1. Experimental Setup

**Figure 1:**
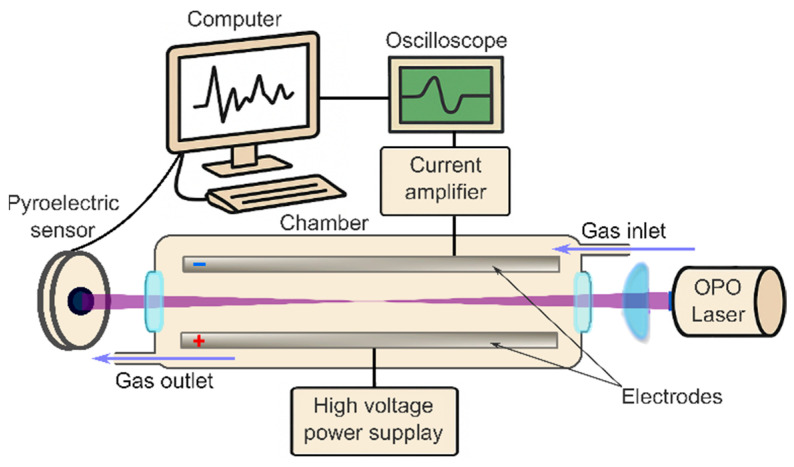
Block diagram of the gas-MEES experimental setup.

A custom-built gas-phase Multiphoton Electron Extraction Spectroscopy (gas-MEES) system was constructed to enable the detection of trace molecular species in gaseous samples under ambient conditions. A schematic overview of the setup is presented in Figure 1. The core measurement chamber, enclosed within a Faraday cage to minimize electromagnetic interference, comprised two stainless steel electrodes (22 cm in length and 2.5 cm in width) separated by a variable gap of 1–2 cm. A high-voltage power supply (Model PS325, Stanford Research Systems, Sunnyvale, CA, USA) was used to apply an electric field of up to 5 kVcm^−1^ across the electrodes. One electrode was grounded and connected to a current preamplifier (DLPCA-200, FEMTO Messtechnik GmbH, Berlin, Germany), which, in turn, interfaced with a digital oscilloscope (Picoscope 3000 series, Pico Technology, Eaton Socon, UK). The oscilloscope was triggered using the internal synchronization pulse of the laser system.

The carrier medium used was ambient air, supplied via the laboratory’s compressed air system.

The laser source was a solid-state optical parametric oscillator (OPO) system (NT 342/3/UVE, EKSPLA, Vilnius, Lithuania), providing tunable ultraviolet (UV) pulses within the 192–410 nm range at a spectral resolution of 0.02 nm. The system operated at a pulse repetition rate of 10 Hz with a pulse duration of approximately 2 ns. Pulse energies ranged between 0.2 mJ and 2.5 mJ, and the energy at each wavelength was monitored using a pyroelectric sensor (PE-10-SH-V2, Ophir Laserstar, Jerusalem, Israel). The laser beam entered the chamber through a 1-inch UV-grade quartz window and was gently focused using a plano-convex lens with a focal length of 30 cm. This long focal length was specifically selected to achieve adequate field strength along the beam path while avoiding plasma formation in the gas phase.

The measurement setup allows for a continuous flow of the analyzed air, with entrance at one end and exit on the other. The rather small volume of the chamber (ca. 0.4 L) allows for the fast replacement of the analyzed gas (ca. 1 s at 24 L min^−1^ flow), which is suitable for online measurements. 

### 2.2. Signal Acquisition and Data Processing

**Figure 2:**
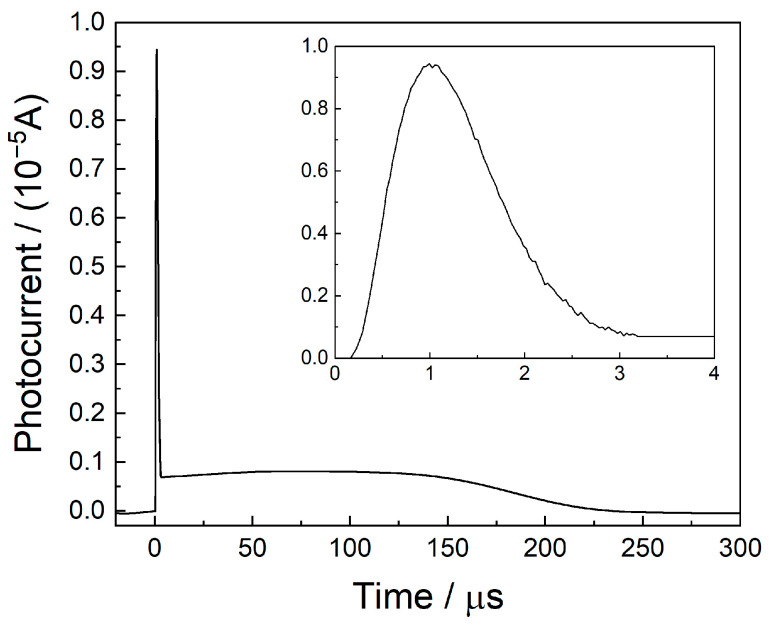
The waveform of current as a function of time.

The slightly focused UV laser beam passes between the two long electrodes and ionizes the gas molecules in a multiphoton process. This results in a photoelectron and a cation, which are separated under the high electrical field between the electrodes. The light electrons are accelerated towards the anode, and they reach the anode after elastic collisions or electron transfers. This occurs during the first ca 3 μs after the laser pulse. A part of the electrons is entrapped by gas molecules of high electron affinity, such as O_2_. The negatively charged molecules move at a slower speed and eventually reach the anode (at a later time), between ca. 3 μs and 200 μs. The cations simultaneously move towards the cathode. The waveform of the current between the electrodes as a function of time is shown in Figure 2, which shows two distinct regimes: the sharp peak due to the electrons and the broad and long one, due to the movement of heavy charged species. 

The broad and long peak results from currents due to a variety of processes that take place after the ionization of the gas molecules. These include the (a) drift of O_2_^−^ (or other negatively charged species) towards the anode, (b) drift of the cation towards the cathode, (c) fragmentation of the initial cation and migration of the fragment baring the positive charge, (d) collisional charge transfer and migration of the new charged species, etc. All the above result in a wide peak, as presented in Figure 2.

Note that the currents are a result of the migration of both negative and positive charges, and they are measured continuously from the time of ionization until they reach the electrode. The currents are recorded during their migration, due to the mirror charges they induce in the electrodes [31]. The resulting transient currents were amplified by the low-noise current amplifier and recorded in real time. 

In MEES, the total photocharge (the integral of the waveform) is calculated; therefore, the specific processes taking place are irrelevant. Nevertheless, the total photocharge depends on the voltage applied between the electrodes. This is because of two main reasons: at higher electrical fields, the probability of charge recombination is lower, and the charge collection efficiency is higher. In other words, under lower electrical fields (e.g., 300 V cm^−1^), some of the charges reach the chamber’s walls instead of the electrode and are practically lost. At electrical fields of ca 5 kV cm^−1^, the collection efficiency approaches 1.

While integration over the sharp peak is straightforward, integration over the whole waveform requires careful inspection of the integration limits. These may depend on the mass of the ionized molecules. Integration over a too-wide range accumulates noise and may result in noisy spectra. Therefore, in many cases, using the first sharp peak is sufficient, although it slightly compromises the sensitivity. 

For each laser pulse, both the integrated current, i.e., the photocharge, and the laser energy are simultaneously recorded. Since laser power fluctuations (of ca. 10%) are unavoidable, the photocharge is normalized by the laser pulse energy. 

All aspects of system operation, data collection, and signal processing were automated through a custom software suite developed in VB.NET (version 17.5.2).

### 2.3. Analytes

The following BTEX analytes were used: benzene (Spectrum, New Brunswick, NJ, USA), toluene (Mallinkrodt, Deventer, The Netherlands), ethylbenzene (Thermo Fisher Scientific, Waltham, MA, USA), and o-, m-, and p-xylene (Sigma Aldrich, St. Louis, MO, USA). The procedure for their preparation at a well-defined concentration in air is described below.

### 2.4. Sample Preparation

Polytetrafluoroethylene (PTFE) calibrated permeation tubes (PT) containing the analytes were prepared using the standard method [32]. The concentration of the analytes in air was established by controlling the airflow rate (2 to 28 liters per minute) and the known permeation rate of each individual tube at a given temperature (in the range of ng min^−1^). 

All flow rates used are sufficient to allow unperturbed diffusion-controlled permeation of the analytes through the tube walls.

## 3. Results and Discussion

### 3.1. Spectra of Ambient Air

**Figure 3:**
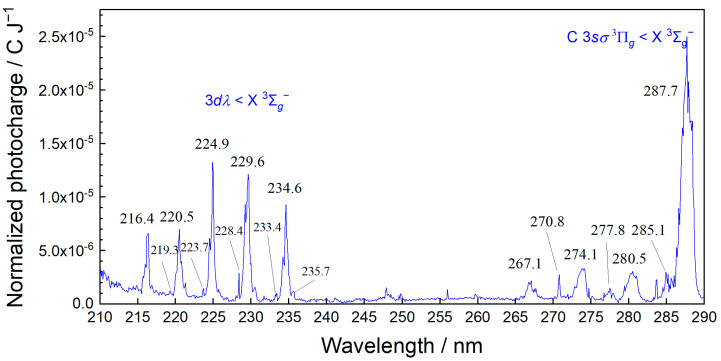
MEES spectrum of ambient air. Some of the known oxygen lines are marked.

MEES is one of the most sensitive spectroscopies, and ambient air provides considerable measurable signals. The MEES spectrum of lab air is shown in Figure 3. The large peaks correspond to ambient oxygen, which is ionized in a 2 + 1 photon absorption [33]. The very small peaks are related to minor impurities due to natural air organic constituents and contaminations in the pipes. In this study of BTEX sensing, we selected the spectral range from 230 to 280 nm in order to prevent interferences with the oxygen peaks. In this range, BTEX compounds have numerous characteristic peaks. We left one of the oxygen peaks (235 nm) in the range for reference. Note that the ionization potential of oxygen (13.62 eV) is much higher than those of BTEX compounds (8.44–9.24 eV); therefore, the MEES spectrum of oxygen is typically much lower than BTEX spectra. In the selected wavelength range (230 to 280 nm), two photons are sufficient for the ionization of BTEX compounds, while three photons are needed for the ionization of oxygen. The ionization cross section of a three-photon process is much lower than that of a two-photon process. Nevertheless, in view of the concentration difference, when performing quantitation of BTEX compounds in the sub-ppb range, the oxygen peaks must be considered.

### 3.2. BTEX Identification

**Figure 4:**
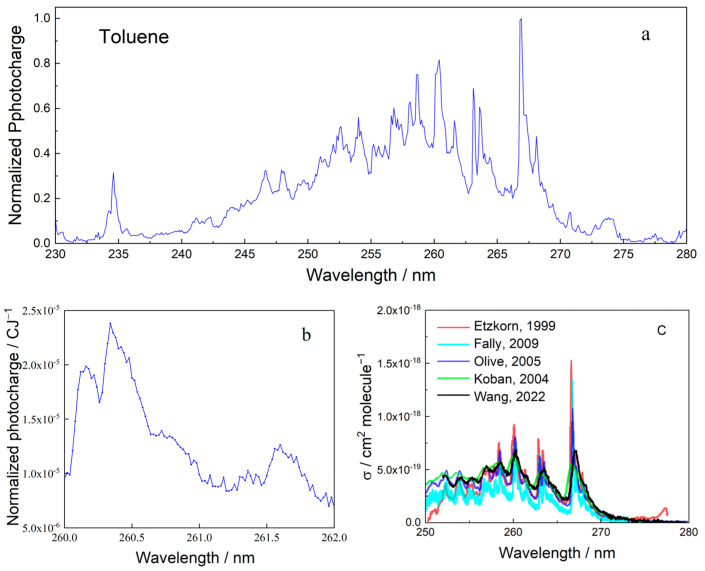
(**a**) MEES spectrum of toluene in air at 0.1 nm resolution. (**b**) A segment of the MEES spectrum of toluene at a resolution of 0.02 nm. (**c**) Incoherent broadband cavity-enhanced absorption spectrum of toluene (black) and spectra measured using other methods. Data reproduced from [33,34,35,36,37] and text reformatted for clarity.

Full MEES spectra were taken of all BTEX materials, with benzene spectra being previously reported [21]. All spectra were measured at concentrations below 100 ppb. The spectrum of toluene in the range from 230 to 280 nm, measured at a resolution of 0.1 nm, is shown in Figure 4a. The actual resolution of our instrument is much better and can be as low as 0.02 nm; however, presenting such details in a wide range is beyond the printer/screen resolution. A segment of the toluene spectrum, the peak in the interval 260 to 262 nm, is presented in Figure 4b. These spectra demonstrate the quality of MEES spectra in terms of the number of peaks and analytical information. For comparison, the spectrum of the same compound, recently (2022) measured using incoherent broadband cavity-enhanced absorption spectroscopy, is presented in Figure 4c [33]. This figure also shows four additional spectra of toluene measured using other methods, all measured offline. The comparison shows that MEES provides not only more spectral details, but also that it does so online.

**Figure 5:**
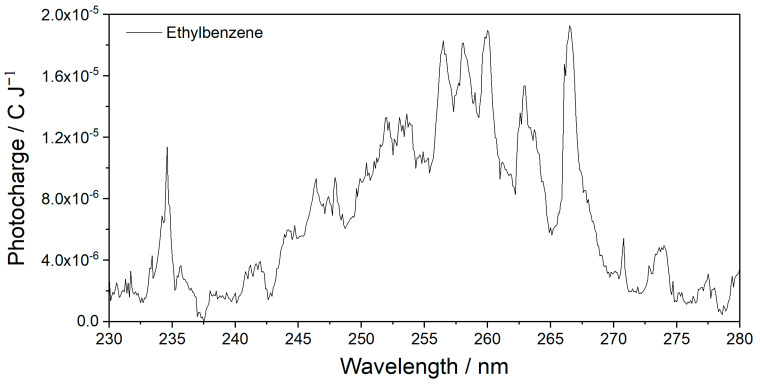
MEES spectra of ethylbenzene; 0.1 nm resolution.

The MEES spectrum of ethylbenzene, measured in the same range at a resolution of 0.1 nm, is presented in Figure 5. Also in this case, the rich spectral details indicate the high potential of using MEES for material fingerprinting and identification. 

**Figure 6:**
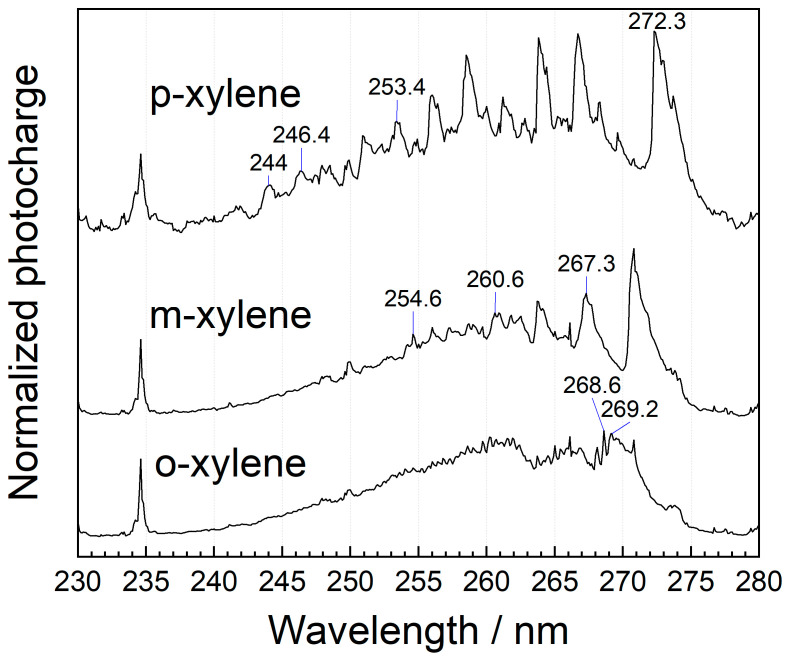
Gas-phase MEES spectra of xylene isomers in air at a wavelength range from 230 to 280 nm, measured at 0.1 nm resolution. Some distinctive peaks are marked.

MEES can also be used for distinction between o-, m-, and p- isomers of xylene. The corresponding MEES spectra in the range from 230 to 280 nm, measured at a resolution of 0.1 nm, are shown in Figure 6. The best distinction should be based on chemometric analysis; however, there are some unique peaks that can also be utilized for o-xylene at 268.6 and 269.2 nm wavelengths. For m-xylene, the distinctive peaks include 354.6, 260.4, and 267.2 nm. p-xylene can be recognized by its peaks at 244.0, 246.4, 253.4, 272.3 nm. 

The interpretation of MEES spectra is not as straightforward as that of absorption spectra, because MEES is the result of a multiphoton process involving higher energy levels. The peaks represent resonant transitions from the ground state to electronic and vibronic excited states. Unlike the absorption spectrum, excitations that are not followed by the absorption of additional photons are not represented in the MEES spectrum. For most BTEX molecules, the higher energy levels have not yet been calculated; therefore, peak assignment in MEES spectra cannot be provided. In the current measurement range, only some of the toluene MEES peaks can be assigned to known spectral transitions in the vapor phase [38,39].

### 3.3. BTEX Quantification

**Figure 7:**
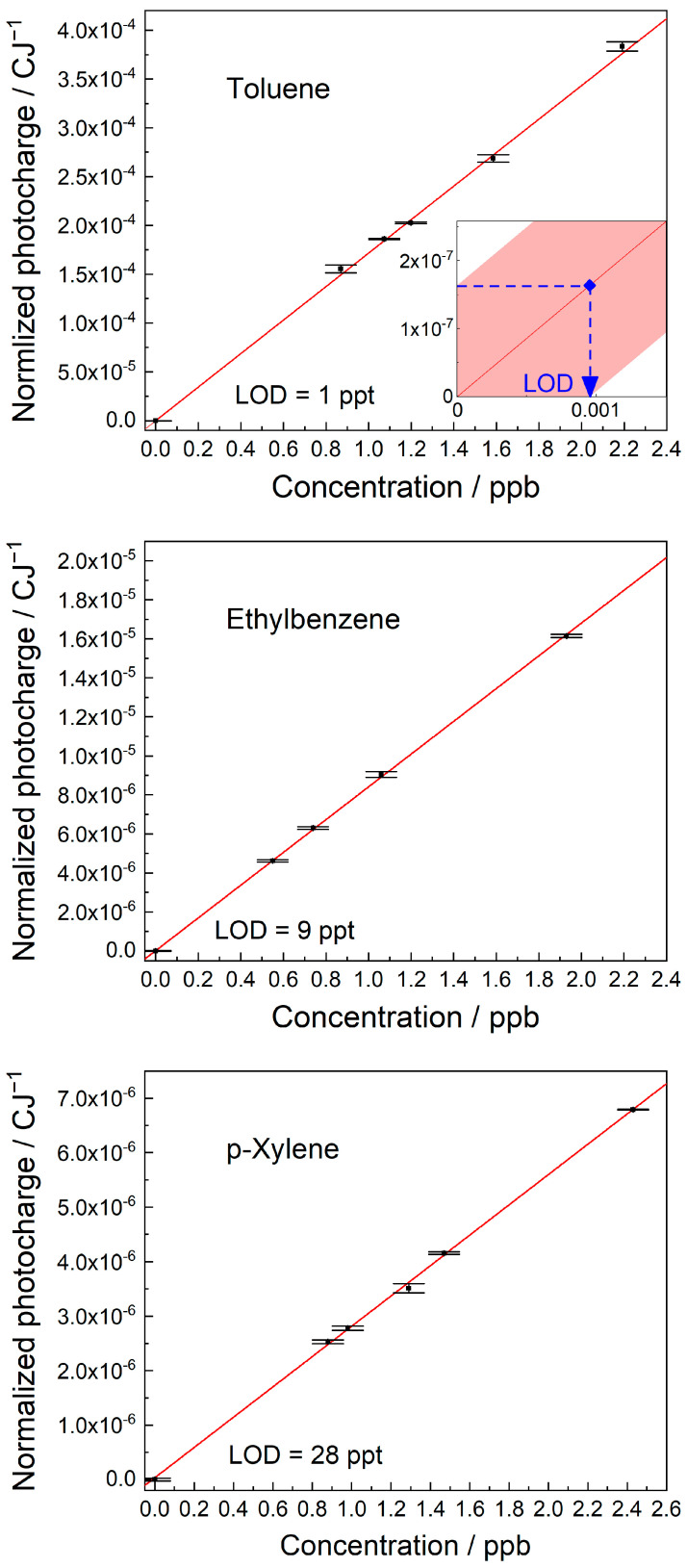
Calibration plots for (**top**) toluene, (**middle**) ethylbenzene, and (**bottom**) p-xylene. The LODs based on 95% confidence intervals are presented. Illustration of the LOD calculation is shown in the inset.

The quantification of BTEX compounds in air was based on calibration plots of MEES signal intensity against concentration. Samples at a given concentration were prepared as described in the experimental session. The corresponding MEES signals were recorded at the highest peak in the range. The limits of detection (LODs), based on 95% confidence intervals, were calculated [40]. The calibration plots are shown in Figure 7. 

The calibration plot of toluene was measured at 266.86 nm and resulted in a LOD of 1 ppt. Ethylbenzene was measured at 266.54 nm and resulted in a LOD of 9 ppt. p-xylene was measured at 273.72 nm and resulted in a LOD of 28 ppt. Benzene was measured at 259 nm and resulted in a LOD of 20 ppt [21]. The calibration plots of all BTEX compounds were linear. 

### 3.4. Additivity 

Often, a mixture of BTEX compounds can be found in the air. Thus, testing for the additivity of MEES signals is relevant. Interferences can be suspected due to several reasons, such as the following: (a) differences in the electron affinity of the molecules and/or their fragments affect the probability of capturing electrons on the way to the anode; (b) differences in the molecular mass cause a difference in cations’ flight time, which is related to the probability of losing the charge upon diffusion to the vessel wall. Since these processes depend on irradiation wavelength, one can suspect that the additivity is wavelength-dependent. Moreover, signal additivity can also be a function of concentration. 

The signal additivity was tested for a mixture of benzene and toluene in the ppb range. For this purpose, MEES spectra of each compound alone were measured, and then the spectrum of the corresponding mixture was taken in the same spectral range. The concentrations were kept constant in all measurements. Finally, the mathematical sum of the spectra of the individual compounds was compared to the spectrum of the mixture. In view of the potential interferences, we integrated the signals along the whole waveform. This slightly increases the noise level but might reveal interesting interferences. 

**Figure 8:**
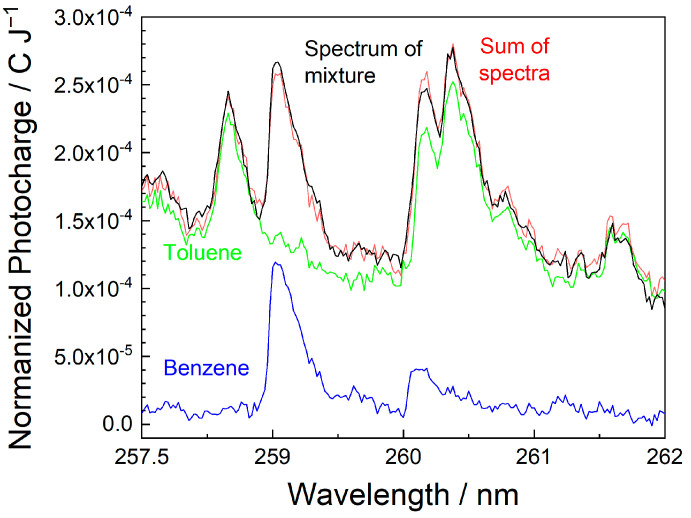
Spectra of benzene (blue), toluene (green), their mathematical sum (red), and the spectrum of the mixture (black). All spectra were taken between 258 and 263 nm at a resolution of 0.02 nm.

The results are shown in Figure 8. The spectra were measured at the highest resolution (0.02 nm) in the range of 258–262 nm, where several peaks of both compounds are present. The toluene signals are higher (as expected, due to its lower ionization potential). Generally, the mathematical sum of the individual spectra somewhat overlaps the spectrum of the mixture, which indicates good additivity. Some minor deviations are observed, especially at wavelengths where both compounds are at resonance; however, the gap is of the order of only 4%. The deviations at other wavelengths are lower and within the experimental error range. The conclusion is that at the ppb concentration range, interferences due to the mixture of compounds are minor and can be avoided by selecting proper wavelengths. Nevertheless, these effects are interesting and should be further investigated. 

### 3.5. Online Measurements

**Figure 9:**
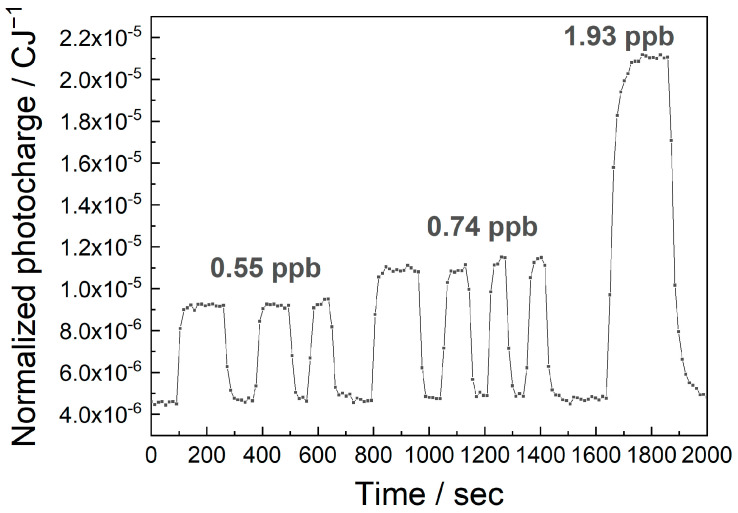
An online measurement of the normalized photocharge at 266.86 nm, upon changing the concentrations of ethylbenzene in air.

The MEES setup is based on continuous measurements while the tested gas flows through the chamber; thus, it allows for real online measurements. The online performance and the response times were demonstrated in an experiment, where the permeation tubes were periodically placed in the flow and removed. The results for various concentrations of ethylbenzene are shown in Figure 9.

The first observation is the repeatability of the measurements. Three replicates of the same concentration resulted in good repeatability of the MEES signals. The second observation is that the response time is good. The theoretical response time in MEES is of ca 1 s, because the laser fires at 10 Hz and we integrate over 10 pulses. The real response time is longer because we had to fill the chamber (ca. 400 mL) with the fresh sample. At a flow rate of 20 Lmin^−1^, this takes 4 s. This time can be shortened by using a smaller chamber or a higher flow rate. In the above measurement, the response time was actually limited by the time needed to remove the permeation tube and return it to the flow.

## 4. Conclusions

In this study, we presented the MEES system as a new and effective method for the monitoring of BTEX compounds in air, with potential applications in environmental protection and analytical chemistry. The system demonstrated rapid response, high sensitivity, and selectivity, making it suitable for online detection and quantitation of BTEX compounds.

Individual, distinctive spectral fingerprints were obtained for benzene, toluene, ethylbenzene, and each xylene isomer, enabling reliable compound identification. The system achieved sub-ppb detection limits, exhibited a good linear relationship between signal intensity and concentration, and maintained a high signal-to-noise ratio. 

Furthermore, we demonstrated the additivity of MEES signals in this concentration range, which may allow for the construction of linear multivariate analysis models, such as PCR and PLS, for resolving BTEX mixtures. The development of proper chemometric algorithms for handling complicated mixtures and matrices is planned.

The MEES system offers several advantages over existing technologies, including fast analysis times, low maintenance requirements, low detection limits, and online monitoring capabilities—all of which position it as a competitive alternative in BTEX analysis. The current limitations include the need for an OPO laser, which at this stage is costly (but much less than GCMS), and the lack of libraries of many VOCs. However, both drawbacks are solvable.

In this study, all calibration plots were based on the signals integrated over the first 3 μs after the laser pulse, which are due to the electrons. This allows simplicity of data analysis, avoiding the handling of the complexity of the signals due to the heavier masses reaching the electrodes. Nevertheless, the signals at longer times might include additional information, which may result in better analytical performance. Investigating the information from longer times (3–300 μs) is planned. 

This research was carried out under controlled laboratory conditions, and future work should test field performance under real ambient air environments. Among the many challenges are potential interferences at high concentration ranges, matrix effects, high humidity, and instrumental stability. 

## Data Availability

The original contributions presented in this study are included in the article. Further inquiries can be directed to the corresponding authors.
